# A cost minimisation analysis of a telepaediatric otolaryngology service

**DOI:** 10.1186/1472-6963-8-30

**Published:** 2008-02-04

**Authors:** Cathy Q Xu, Anthony C Smith, Paul A Scuffham, Richard Wootton

**Affiliations:** 1 Centre for Online Health, University of Queensland, Level 3 Foundation Building, Royal Children's Hospital, Herston, Queensland 4029, Australia; 2 School of Medicine, Griffith University, Logan Campus, Meadowbrook, Queensland 4131, Australia

## Abstract

**Background:**

Paediatric ENT services in regional areas can be provided through telemedicine (tele-ENT) using videoconferencing or with a conventional outpatient department ENT service (OPD-ENT) in which patients travel to see the specialist. The objective of this study was to identify the least-cost approach to providing ENT services for paediatric outpatients.

**Methods:**

A cost-minimisation analysis was conducted comparing the annual costs of the two modes of service provided by the Royal Children's Hospital (RCH) in Brisbane. Activity records were reviewed to analyse volume of activity during a 12 month period in 2005, i.e. number of clinics, duration of clinics, number of consultations via telemedicine and in outpatient clinics, diagnoses, and travel related information. A sensitivity analysis was conducted using factors where there was some uncertainty or potential future variation.

**Results:**

During the study period, 88 ENT consultations were conducted via videoconference for 70 patients at Bundaberg Base Hospital. 177 ENT consultations were conducted at the RCH for 117 patients who had travelled from the Bundaberg region to Brisbane. The variable cost of providing the tele-ENT service was A$108 per consultation, compared with A$155 per consultation for the conventional outpatient service. Telemedicine was cheaper when the workload exceeded 100 consultations per year. If all 265 consultations were conducted as tele-ENT consultations, the cost-savings would be $7,621.

**Conclusion:**

The cost-minimisation analysis demonstrated that under the circumstances described in this paper, the tele-ENT service was a more economical method for the health department of providing specialist ENT services.

## Background

Delivering specialty care to children living in the vast region of Queensland is challenging and often requires patients to travel to tertiary centres for specialist consultations. In some circumstances, specialists travel to regional hospitals to conduct outreach clinics. The cost to families and the healthcare system is substantial. For example, the State Government's health department in Queensland (Queensland Health) provides financial assistance to patients who need access to specialist medical services, through the Patient Travel Subsidy Scheme. In the 2005–2006 financial year, $28 million was spent on patient travel [1].

Ear, nose and throat (ENT) disorders are common and represent a large proportion of healthcare problems in children. Consequently, there is a high rate of referrals to ENT specialty clinics [2-5]. In North Queensland, the prevalence of childhood ENT problems, especially otitis media, is extremely high (66%–95%) among aboriginal children [6-8]. The Royal Children's Hospital (RCH) in Brisbane is one of two major paediatric tertiary referral hospitals which service Queensland. In 2005, there were 4,819 ENT outpatient department consultations (OPD) and 1,980 ENT inpatient admissions to the hospital.

**Figure 1:**
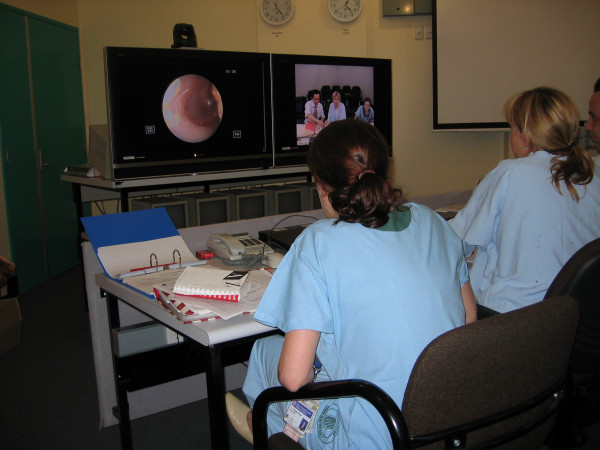
**Tele-ENT clinic in progress. The specialist is able to view video-otoscopic images during a videoconference appointment**.

Telemedicine has been used for the pre-screening of potential ENT surgical patients as part of a research project facilitated by the University of Queensland's Centre for Online Health (COH) [9]. Since 2003, patients in selected regional areas have been referred by their local primary healthcare providers to a telemedicine clinic at their local hospital. Instead of travelling to Brisbane to see the specialist, an ENT consultation is conducted via videoconference. During the videoconference appointment, an ENT examination is carried out using a video-otoscope connected to the videoconference system. The specialist is able to view real-time ENT images at a bandwidth of 384 kbit/s as well as hearing test results and X-rays transmitted via a document camera. Connected at a distance by a videoconference system, the specialist can discuss the history and clinical findings with the patient/family and the local paediatrician in order to make a decision about diagnosis and clinical management (Figure 1). A coordinator based at COH travels to the regional site and provides technical support during each clinic session.

**Figure 2:**
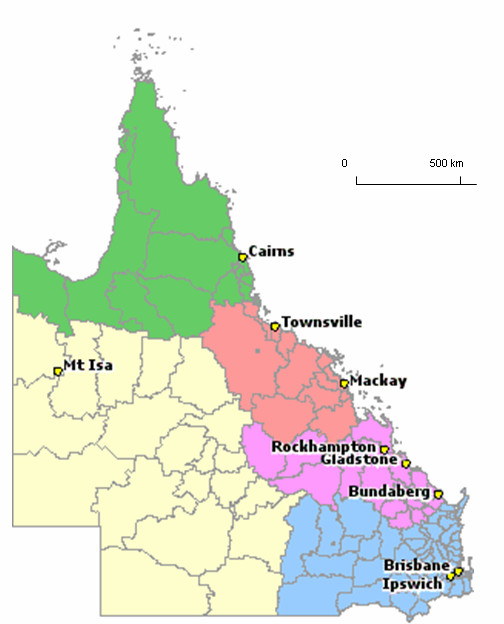
Map of Queensland.

Bundaberg is the region where the pilot tele-ENT work has been carried out. Bundaberg is a coastal region situated approximately 385 km north of the Brisbane (Figure 2). The population of Bundaberg and surrounding area is about 74,000 [10]. In 2005, the RCH conducted 177 conventional outpatient ENT (OPD-ENT) consultations for children referred from Bundaberg. In addition, a total of 88 ENT consultations were conducted by telemedicine.

There are very few studies which have compared the clinical outcomes and costs of telemedicine services to the costs of conventional outpatient services; there are almost none in the area of tele-ENT. Pedersen first reported the use of endoscopic cameras and videoconferencing systems in Norway [11,12]. Tele-ENT has also been described as a useful application in military medicine [13]. Previous work done in Queensland demonstrated the feasibility of videoconferencing for the assessment of otolaryngology conditions during the pre-screening of potential surgical patients. The study described potential savings from the use of telemedicine and suggested that further studies were required on the cost-effectiveness [9]. The objective of the present study was to identify the least-cost approach to providing ENT services for paediatric outpatients in a regional town in Queensland.

## Methods

We conducted a cost-minimisation analysis to compare the cost of the tele-ENT service in Bundaberg to the cost of providing the conventional RCH outpatient service for patients travelling from Bundaberg. We analysed the annual cost of the tele-ENT service for the 2005 calendar year. This was compared with the cost of the conventional outpatient service during the same 12-month period. The outcomes of the consultations were assumed to be the same whether delivered face-to-face or by telemedicine.

Study subjects were:

A. Patients who attended tele-ENT clinics in Bundaberg for their appointments. These patients were referred by the paediatrician who participated in the tele-ENT clinics.

B. Patients who travelled to Brisbane and visited OPD-ENT clinics at RCH for their regular appointments. They were referred by other physicians in Bundaberg.

ENT service information was obtained from the RCH information system and the telepaediatric service activity records maintained by the COH. Clinical information was collected from RCH medical records. Travel costs and reimbursement information was obtained from the Bundaberg Base Hospital Travel Office and from the Patient Travel Subsidy Scheme (PTSS).

The following data were collected: number of clinics, duration of the clinic, number of patients and consultations via telemedicine, number of patients visiting RCH and outpatient consultations provided, diagnoses, travel distance and travel mode (e.g. by car, rail or air), and travel reimbursement information.

### Cost Minimisation Analysis

We calculated the fixed and variable costs of providing the ENT services using a costing model similar to other reported studies [14-19]. Tele-ENT service costs were classified as either fixed or variable costs. Fixed costs are those that are independent of the number of patients using the service, such as equipment and facilities. Variable costs are the associated costs of conducting each consultation. All costs were described in 2005 Australian dollars (A$1 ≈ US$0.80).

Fixed costs were a video-otoscope (Flexiscope Microvision ENT Camera, Inline Systems) and a document camera used to transmit images of hard copy documents such as hearing tests and X-rays. Annual equivalent costs were calculated over a period of five years using an annual discount rate of 5%. Since both the RCH and the regional centre had existing videoconferencing equipment and ISDN lines (for other telehealth services and education programmes), the proportion of the fixed costs of these items ascribed to ENT would have been very small and therefore they were ignored. Facility costs were also excluded as both services utilized existing hospital infrastructure.

Variable costs were based on the standard charges and rates in Queensland. These included staff salaries and travel costs, ISDN line charges and patient travel costs. The time and duration of a consultation depends on the sub-specialty and nature of the consultation. The average tele-ENT consultation time per patient was about 10 minutes, i.e. it was similar to an outpatient face-to-face ENT consultation time. Therefore, the cost of the ENT specialist's time was about the same, regardless of mode of consultation, and was excluded.

Patients who were referred to the RCH for outpatient consultations were eligible for reimbursement for part of their travel cost through the PTSS. The Bundaberg Hospital travel office reported that most patients who visited RCH travelled by rail in 2005. The cost calculation for travel was therefore based on the rail fare. For an outpatient visit, the patient/family was funded for a return trip and accommodation for one night. However, only some families lodged claims or made travel arrangements through the hospital travel office in Bundaberg. Therefore, we calculated the annual costs to the health department based on the number of consultations conducted during 2005, rather than PTSS bookings or claims through the travel office. Additional costs to the family, such as time off work, parking, fuel and meals were not analysed during this study.

Because this study was designed as a cost-minimisation analysis, the threshold (break-even point) at which the tele-ENT service became less costly than the OPD-ENT service was estimated. A sensitivity analysis was conducted using factors where there was some uncertainty or potential future variation. These factors were related to the cost and usage of the video-otoscope, ISDN line charges and the proportion of patients who utilised the PTSS. The tele-ENT project was approved by the appropriate ethics and hospital committees. Ethics permission was not required for the cost analysis.

## Results

In 2005, nine tele-ENT clinic sessions were conducted and a total of 88 ENT consultations were provided via videoconference for 70 patients at Bundaberg Base Hospital. The average number of consultations was 7 per month over the 12-month period, ranging from 7 to 13 consultations each month (excluding case discussion sessions when patients were not present). The average number of consultations via telemedicine was 1.3 per patient over the 12-month study period (Table 1).

**Table 1:** Tele-ENT and OPD-ENT activity involving patients from Bundaberg (2005)

	**Tele-ENT**	**OPD-ENT**
Number of patients	70	117
Number of consultations	88	177
Number of consultations per patient	1.3	1.5

**Figure 3:**
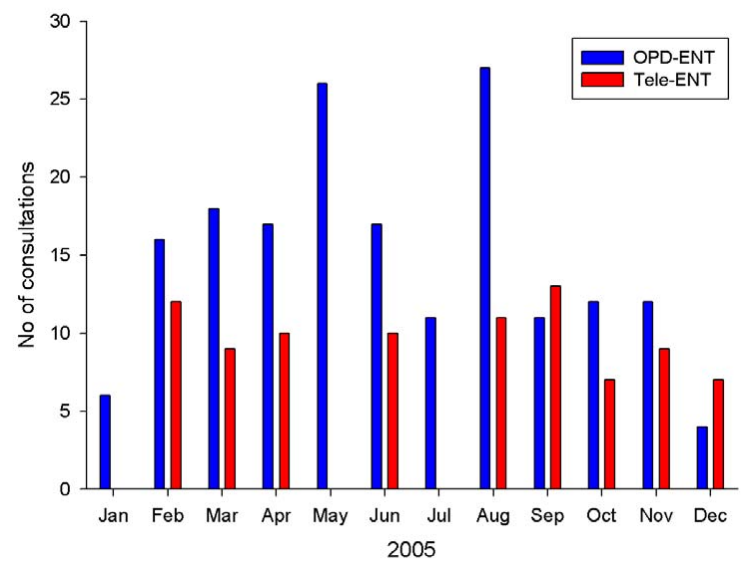
ENT clinical activity in 2005 (both modalities).

The conventional OPD-ENT service provided 177 face-to-face consultations for 117 patients who had travelled from Bundaberg. The average number of consultations in this group was 15 per month, ranging from 4 to 27 consultations each month. The average number of consultations per patient was 1.5 in 2005 (see Table 1, Figure 3).

### Clinical categories

Medical chart reviews confirmed that the types of cases diagnosed during the tele-ENT and OPD-ENT clinics were similar (Table 2). The main diagnoses were otitis media, recurrent tonsillitis, obstructive sleep apnoea, grommets review, hearing loss, allergic rhinitis and cleft palate reviews. We found that there were substantial differences in the proportion of certain conditions diagnosed in each group, which we suspect was mainly because subjects were sorted according to mode of consultations rather than actual clinical conditions.

**Table 2:** Clinical findings (diagnosis) reported during the specialist examination*

	**Tele-ENT**		**OPD-ENT**	
**Diagnoses**	**Number of conditions**	**Proportion (%)**	**Number of conditions**	**Proportion (%)**

Otitis media	32	31	99	43
Recurrent tonsillitis	23	23	34	15
Obstructive sleep apnoea	12	12	27	12
Rhinitis	10	10	5	2
Other	24	24	61	27
Normal	1	1	4	2

**Total**	**102**	**100**	**230**	**100**

* Note: some patients had multiple conditions

### Tele-ENT service costs

The service was provided in pre-existing paediatric telehealth facilities at both the RCH and the Bundaberg Base Hospital. The initial cost of the video-otoscope was $15,000 and the cost of a document camera was $5,000. Assuming these pieces of equipment both have a life of five years, and using a discount rate of 5%, the annual equivalent cost of the equipment was $4,620 (Table 3).

**Table 3:** Summary of costs for the tele-ENT and OPD-ENT service

	**Tele-ENT ($)**	**OPD-ENT ($)**
Fixed costs*		
Equipment – Video-otoscope	3465	0
Document camera	1155	0
		
*Sub total*	*4620*	*0*
		
**Variable costs**		
Paediatrician	2700	0
Technician	2160	0
Technician's travel costs	2700	0
ISDN line charges	1980	0
Patient/family travel costs (child and parent fare by rail)	0	16,744
Patient/family accommodation	0	10,620
*Sub total*	*9540*	*27,364*
		
**Total cost**	**14,160**	**27,364**
**Variable cost per consultation**	**108**	**155**

*Annual equivalent cost depreciated over 5 years at 5%

Variable costs included a local paediatrician's salary at an average rate of $150 per hour for a duration of 2 hours per clinic. Other variable costs included a technician's salary of $30 per hour for one working day (8 hours), the technician's return airfare and ISDN line charges at a rate of $110 per hour. Table 4 lists the total variable costs of the tele-ENT service.

**Table 4:** Variable costs for the tele-ENT service

**Variable costs**	**Rate**	**Clinic duration**	**Number of clinics**	**Annual costs ($)**
Paediatrician's salary	$150/hour	2 hours	9	2700
Air travel	$300/trip	1 return trip	9	2700
Technician ($30 × 8 hr)	$240/day	8 hours	9	2160
ISDN line charges	$110/hour	2 hours	9	1980
Total variable cost				**9540**

The average variable cost per consultation was $108 using the tele-ENT service. The annual cost of providing 88 consultations was $14,160. The estimated total annual costs would have been $33,348 if all ENT consultations (265) had been conducted via telemedicine at a variable cost of $108 each plus fixed costs of $4,620.

### OPD-ENT service costs

The Queensland Health PTSS reimbursement for Bundaberg patients was $94.60 ($30.80 for a child plus $63.80 for the escort) for travel by rail to Brisbane. Accommodation assistance was provided to the patient and an approved escort of up to $30 per person per night. Thus, for a paediatric OPD appointment, the accommodation cost was $60. For the 177 OPD consultations conducted in 2005, the estimated costs of travel by train and accommodation were $16,744 and $10,620 respectively. Thus, the average estimated travel cost per OPD consultation was $155. Assuming all ENT consultations conducted in 2005 were conducted in outpatients (OPD-ENT), the estimated annual travel costs would have been $41,075 (i.e. 265 consultations at $155 each).

### Threshold

**Figure 4:**
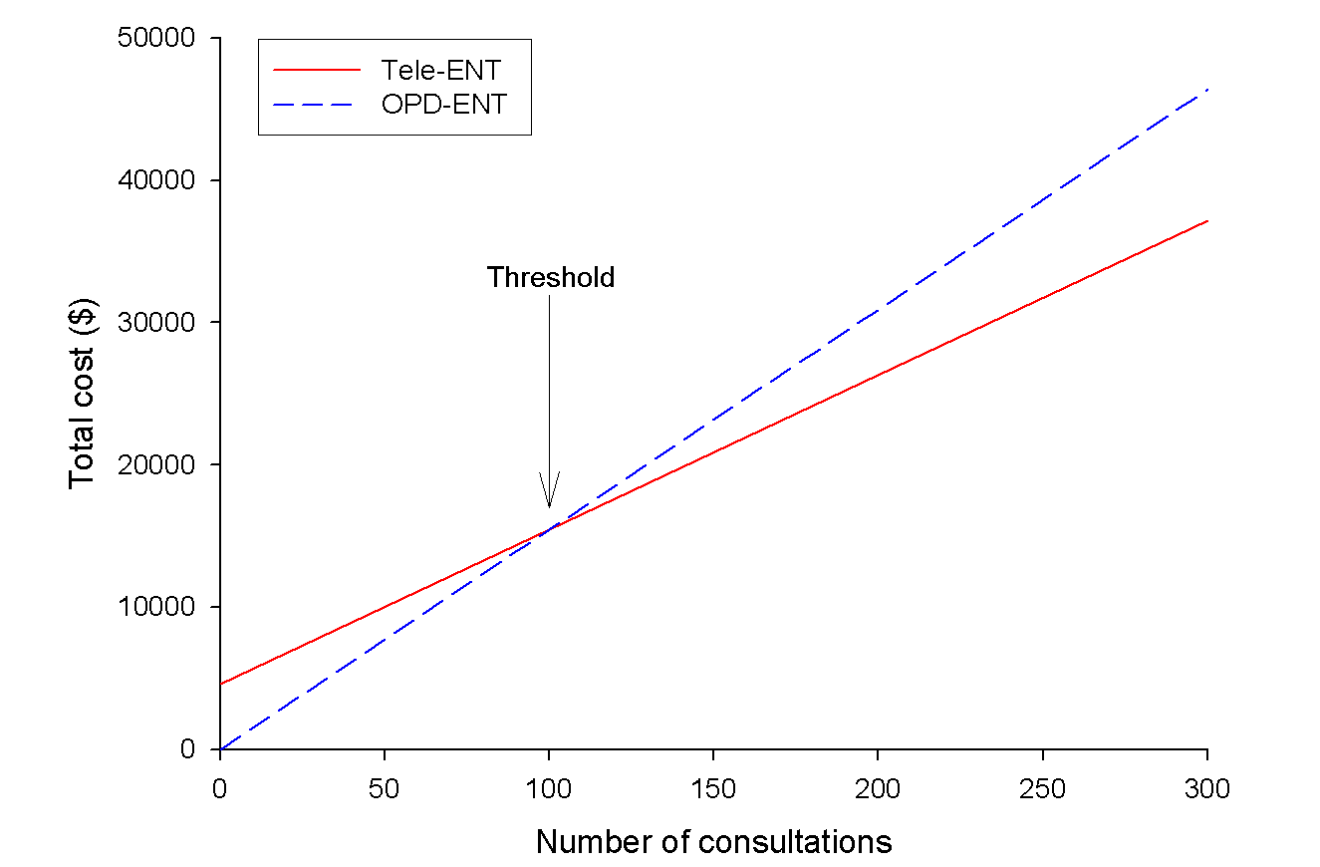
Threshold analysis.

During the study, the total cost for an OPD consultation was $155 per consultation and for tele-ENT the cost was $161 per consultation. The variable costs were less for tele-ENT compared with OPD ($108 vs $155). Thus, tele-ENT was slightly more costly than OPD due to the fixed costs for the video-otoscope and document camera. We calculated the threshold (break-even point) at which the tele-ENT service became less costly than the OPD-ENT service (Figure 4). The threshold point was 100 consultations. That is, additional tele-ENT consultations above this threshold were cost-saving for the health department compared with outpatient consultations. The difference between conducting all 265 consultations conventionally and all 265 by telemedicine was a cost-saving of $7,621.

### Sensitivity analysis

The sensitivity analysis showed that the expected useful life of the video-otoscope was a key factor; the effect on the results of changes in the life of the instrument was greater than the effect of changes in the initial cost of the instrument. The costs of accommodation and travel were also important factors; when these costs were increased, fewer OPD-ENT consultations were required for tele-ENT to become cost-saving. Similarly, when the costs of ISDN line charges were reduced by 50%, fewer consultations were required for tele-ENT to be cost-saving. The travel costs for a technician had a relatively substantial effect on the result. For example, when these were increased by 50%, 147 consultations would need to be performed by tele-ENT for the service to be cost-saving. However, if a local technician could be employed or seconded on a pro-rata basis, travel costs would reduce towards zero. Under this scenario, tele-ENT becomes cost-saving when 60 consultations are undertaken. The discount rate had a relatively small effect only (Table 5).

**Table 5:** Sensitivity analysis

**Adjusted unit costs**	**Workload (number of consultations) required for threshold to be reached**
Base case	100
Video-otoscope	
cost = $12,000	85
cost = $18,000	115
life = 3 years	144
life = 8 years	75
discount rate = 3%	96
discount rate = 10%	111
Document camera	
cost = $4000	95
cost = $6000	105
life = 3 years	115
life = 8 years	95
discount rate = 3%	99
discount rate = 10%	104
Proportion seeking reimbursement for travel	
90%	150
80%	303
Travel cost	
$115	69
$125	60
Accommodation allowance	
$40	70
$50	54
ISDN line charges	
$1000	81
$3000	134
Technician's travel costs	
$0	60
$1350	75
$4000	147

The above calculations were based on both specialist and regional centres having existing videoconferencing equipment and ISDN telecommunications. If a tele-ENT service was to be introduced to a location that did not have this infrastructure, additional expenditure would be required. To establish a new site, the fixed costs would increase to include basic videoconferencing equipment and installation of telecommunications. We estimate these would cost an annual equivalent of $12,750 over a five-year period (see Table 6).

**Table 6:** Estimated costs of videoconference equipment and telecommunications for a new site

**Fixed costs**	**Provider site ($)**	**Patient site ($)**	**Total ($)**	**Annual equivalent cost ($)^1^**
Videoconference unit	7000	7000	14,000	3234
TV monitor	500	500	1000	230
ISDN installation^2^	885	885	1770	n/a
Video-otoscope	0	15,000	15,000	3465
Document camera	0	5000	5000	1155
Trolley	750	750	1500	346
				
ISDN line rental^3^	2160	2160	4320	4320

**Total**	**11,295**	**20,295**	**31,590**	**12,750**

^1 ^Depreciated at 5% annually with no resale value at the end of 5 years.

^2 ^This is a sunk cost occurring in year one only.

^3 ^3 ISDN lines for 12 month ($60 per line per month).

## Discussion

There is an emerging literature on the utility and efficacy of telemedicine. However, studies of the cost effectiveness of telemedicine practice have been limited. According to a number of systematic reviews, there is a lack of persuasive evidence about whether telemedicine is a cost-effective way of delivering health care [20,21]. The present study suggests that from the perspective of the health service provider, the tele-ENT service is less expensive than the conventional OPD service in existing telehealth facilities as shown in Table 3. The tele-ENT service was cheaper when the workload exceeded 100 consultations per year. This is significant to the region of Bundaberg where 265 ENT consultations took place in 2005. Our finding is consistent with other studies that suggest that with a higher number of telemedicine consultations, greater savings are made by the health service provider [14,17,19].

In the case of Bundaberg, 33% of all ENT consultations were conducted via telemedicine in 2005. If each telemedicine consultation was a direct substitute for an outpatient consultation, then the tele-ENT service was cost-saving. This suggests that potential savings to the health system could be obtained if telemedicine was utilized on a wider scale for paediatric ENT services, particularly for regions with similar patient referral rates and at similar or greater distances from the specialist centre.

In principle, the service could be expanded to other suitable sites without a major increase in fixed costs, especially in locations with existing telemedicine facilities. However, there are a number of factors that need to be considered should tele-ENT services be expanded. These include the availability of ENT specialists, involvement of regional clinical staff and technical support. The service also relies on an adequate telemedicine infrastructure.

Although not formally measured in the present analysis, the family costs of not having to travel to Brisbane to see a specialist should be acknowledged. This is certainly the case for families living in the rural areas where specialist services are lacking. Smith et al (2003) compared the family costs of attending hospital outpatient appointments via videoconference and in person. The results of that economic study suggested that it was much more expensive and inconvenient to travel to the RCH for an outpatient consultation than it was to attend a telepaediatric consultation at a regional hospital [22].

Travel to Brisbane from Bundaberg usually takes 5–6 hours by car or train – one way. From the health department's perspective, tele-ENT cost an additional A$6 per consultation more than OPD during the study period; however, each additional tele-ENT consultation would save the health department $46 because all fixed costs have been covered. In addition, many regional families have no choice but to take time off work to attend an appointment in Brisbane and children are unable to attend school. There are also out-of-pocket expenses which are not reimbursed by the health service such as child care costs, meals and parking fees. If all of these costs were taken into consideration, the overall societal economics of doing telemedicine would have been even more favourable.

Few studies have examined the clinical outcomes of tele-ENT in comparison to OPD-ENT consultations. Several studies have investigated the accuracy of pre-recorded digital images and found over 80% concordance compared with follow-up and real-time consultations [23,24]. There are a number of studies which have compared real-time and store-and forward ENT tele-consultations. These studies found a higher rate of diagnostic accuracy using real-time methods [25,26]. A recent study examined the accuracy of real-time telemedicine for paediatric ENT pre-admission screening. Data on the patient's initial videoconference diagnosis and management strategy were compared to eventual primary diagnosis and patient care. The diagnostic and treatment agreement were both over 90% [27].

There are certain limitations in the present study. We assumed that the clinical outcome from a tele-ENT consultation was similar to that from an outpatient consultation. Also there were substantial clinical variations between the two study groups since the clinical case-mix was not controlled. Finally, it was an observational study and not a randomised controlled trial. Thus the economics of using telemedicine for paediatric ENT assessments will require further investigation before definitive conclusions can be drawn.

## Conclusion

In summary, the present study compared the costs of a tele-ENT service to the costs of providing OPD-ENT services in the conventional manner. The cost analysis was based on the annual activities during 2005, and suggests that savings to the health care system could be made when actual workload exceeded 100 tele-ENT consultations. The variable cost of tele-ENT consultation was less than the cost of travel to Brisbane from a regional area to attend OPD-ENT clinics.

## Competing interests

The author(s) declare that they have no competing interests.

## Authors' contributions

CX and RW conceived the study and conducted the analysis. CX produced the first draft of the manuscript. AS contributed to the analysis, study design, methodology and revisions of the manuscript. PS provided valuable guidance and contributed to the health economic evaluation. All authors contributed to the reviewing and editing of the manuscript. All authors read and approved the final manuscript.

## Pre-publication history

The pre-publication history for this paper can be accessed here:


